# Characterization of siderophores from *Escherichia coli* strains through genome mining tools: an antiSMASH study

**DOI:** 10.1186/s13568-022-01421-x

**Published:** 2022-06-15

**Authors:** Levent Cavas, Ibrahim Kirkiz

**Affiliations:** 1 The Graduate School of Natural and Applied Sciences, Department of Biotechnology, Dokuz Eylül University, Kaynaklar Campus, 35390 İzmir, Türkiye; 2 Dokuz Eylül University, Faculty of Science, Department of Chemistry, 35390 Kaynaklar Campus, İzmir, Türkiye

**Keywords:** Bioinformatics, *Escherichia coli*, Genome mining, Siderophores, Urinary tract infections

## Abstract

Although urinary tract infections (UTIs) affect many people, they are usually a disease observed in women. UTIs happen when exogenous and endogenous bacteria enter the urinary tract and colonize there. Cystitis and pyelonephritis occur when bacteria infect the bladder and the kidneys, respectively. UTIs become much serious if the bacteria causing the infection are antibiotic resistant. Since the pathogenic microorganisms have been adopted to current antibiotics via genetic variations, UTIs have become an even more severe health problem. Therefore, there is a great need for the discovery of novel antibiotics. Genome mining of nonpathogenic and pathogenic *Escherichia coli* strains for investigating secondary metabolites were conducted by the antiSMASH analysis. When the resulting secondary metabolites were examined, it was found that some of the siderophores are effective in UTIs. In conclusion, since the siderophore production in *E. coli* is directly related to UTIs, these molecules can be a good target for development of future pharmaceutical approaches and compounds. Siderophores can also be used in industrial studies due to their higher chelating affinity for iron.

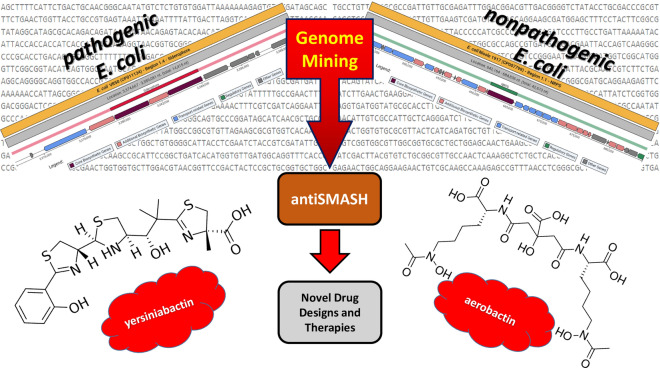

## Key points

- Genome mining on nonpathogenic and pathogenic *E. coli* was studied.
- Comprehensive and comparative analysis of siderophores were investigated.
- The results may open a new gate on the development of new drugs on pathogenic *E. coli*-based diseases.

## Introduction

Urinary tract infections (UTIs) are the most common among infections transmitted by pathogenic *Escherichia coli* strains*.* UTIs have become a global health problem due to the difficulty of detecting antibiotic-resistant pathogens (Ndzime et al. 2021). Almost half of the women and 12% of men have this infection once in their lifetime (Tabatabaie et al. 2022). Due to the hormonal and anatomical changes that occur during pregnancy, women are more likely to get UTIs (Kalinderi et al. 2018). Although there has been no evidence of human-to-human transmission of this infection, consumption of water and food contaminated with *E. coli* can cause outbreaks in communities (Manges et al. 2001). Because UTIs caused by *E. coli* do not show obvious symptoms, they are often seen as a harmless or short-term affliction. If it is not taken seriously, they can cause cystitis, pyelonephritis, and bacteriuria (Foxman 2002). The *E. coli* strains are presented by pathogenic and nonpathogenic characteristics. Nonpathogenic and commensal *E. coli* strains are usually in a symbiotic relationship with its host and have important roles in the human gastrointestinal tract. Therefore, the nonpathogenic strains rarely cause diseases (Bien et al. 2012). However, the pathogenic strains can also colonize the intestines. These strains differ from each other according to whether they contain the gene region responsible for virulence factors. *E. coli* Nissle 1917 (EcN) is a nonpathogenic and commensal *E. coli* strain that benefits humans without any harm. EcN is used for treating diseases such as gastrointestinal and UTIs by competing with pathogens (Schultz 2008; Ou et al. 2016). On the other hand, Beatson et al. (2015) reported that *E. coli* VR50 causes urinary tract infections via gene modifications. UPEC strains contain many genes that produce virulence factors to increase pathogenicity. The more virulence factors an organism expresses, the more serious infections it can cause. The organisms use virulence factors to attach, invade and attack the host. The virulence factors include adhesins, toxins, siderophores, protective polysaccharide coatings, invasins to aid colonization and increase the severity of the infection (Yun et al. 2014; Beatson et al. 2015).

Iron is essential for living organisms as it is a cofactor for many cellular processes. Electron transport system, oxidative phosphorylation, citric acid cycle, superoxide metabolism and DNA/RNA synthesis are among these processes. It is also involved in the production of toxins, antibiotics and siderophores (Fardeau et al. 2011; Negash et al. 2019). The organisms produce siderophores to acquire essential iron from the environment in response to iron deficiency. Siderophores are low molecular weight organic chelators that have a very specific affinity for Fe (III). The iron affinity of the siderophores is so high that they remove iron from molecules, which iron binds to such as ferritin, transferrin and lactoferrin (Ratledge and Dover 2000; Li et al. 2016). Because of this feature majority of the siderophores have gained importance due to their virulence effects on pathogens. In fact, pathogens that produce an excessive number of siderophores are referred to as hypervirulent, whereas pathogens that cannot produce siderophores have lower virulence during infections (Holden and Bachman 2015). Additionally, deletion of genes responsible for siderophore biosynthesis has been associated with reduced virulence in pathogens such as Gram-positive and Gram-negative bacteria (Khasheii et al. 2021). Differentiation of siderophore biosynthesis may impart new or improved properties to the siderophore, causing the bacterium to become more virulent. *E. coli* strains produce four types of siderophores, which are enterobactin, salmochelin, yersiniabactin and aerobactin. Among these siderophores, it was found that salmochelin and yersiniabactin were produced more in UPEC strains (Feldmann et al. 2007; Henderson et al. 2009). Khasheii et al. (2016) found that the irp2 (yersiniabactin) siderophore gene is the most abundant gene in UPEC strains.

There are two pathways for siderophore biosynthesis: non-ribosomal peptide synthetase (NRPS)-dependent and NRPS-independent pathways. Siderophores are peptides that are synthesized by NRPSs, which are modular, multi-domain enzymes. NRPSs are also responsible for the biosynthesis of most of the secondary metabolites (Barry and Challis 2009). The NRPS-independent siderophore (NIS) pathway contains different kinds of synthetases. NIS synthetases perform a single enzymatic reaction. All NIS enzymes carry a N-terminal iron uptake chelate (*IucA/IucC*) domain and have a C-terminal domain related to iron transport or metabolism (Oves-Costales et al. 2009).

Nowadays, natural compounds form the basis of new therapeutic drugs. Microorganisms producing secondary metabolites contain biosynthetic gene clusters (BGCs) in which more than one gene is located close to each other. Therefore, BGCs and the natural compounds they produce have great therapeutic potential. Bacterial, fungal and plant secondary metabolites are pharmacologically effective compounds that are used for developing new drugs (Prihoda et al. 2021). Genome-based drug discovery approach is used to reveal gene clusters that synthesize bioactive compounds and to propose novel therapeutic drugs from these bioactive compounds. At this point, tools such as antiSMASH that identifies BGCs have gained great importance (Mushtaq et al. 2018). When the whole genome sequence of a microorganism is enlightened, it may be a pioneer for secondary metabolite studies (Kim et al. 2017). For this reason, the importance of genome mining studies in secondary metabolite production has been underlined (Albarano et al. 2020). The determination of secondary metabolites is carried out by experimental procedures that vary and require time. However, bioinformatics tools such as antiSMASH provide results quickly. AntiSMASH is a rapid and reliable source for finding gene clusters responsible for the biosynthesis of secondary metabolites (Villebro et al. 2019; Medema et al. 2011). AntiSMASH analysis also give detailed information of the secondary metabolites predicted. Thus, the types of secondary metabolites that cause pathogenicity are revealed (Zotchev et al. 2012). This could lead to novel methods of treating people with diseases such as UTIs. In diseases that are difficult to diagnose such as urinary tract infections, changing the treatment method according to the type of the metabolite will make it easier to get rid of the pathogens that cause the disease.

No scientific publication has so far been reported on the identification of siderophores in UPEC by using antiSMASH technology. The PUBMED Search results are given in Table 1.

**Table 1:** The searched key words of the study (04.04.2022)

Searched Key Words	Items in PUBMED
(Urinary tract infection)	76,193
((Urinary tract infection) AND (siderophore))	200
(((Urinary tract infection) AND (siderophore)) AND (*Escherichia coli*))	121
(((Urinary tract infection) AND (siderophore)) AND (*Escherichia coli*)) AND (antiSMASH)	0

When the whole genome of a microorganism is revealed, the regions related to secondary metabolite production can be predicted. Genome mining has an important place in predicting these metabolites (Villebro et al. 2019; Albarano et al. 2020). As the importance of secondary metabolites becomes clear, genome mining tools begin to be developed. With the increase in genomic data, genome mining tools used for secondary metabolite production has become indispensable (Kim et al. 2017).

The discovery of biosynthetic gene clusters related to siderophore synthesis in pathogenic and nonpathogenic *E. coli* strains by using genome mining technologies was aimed at investigating in the present study.

## Materials and methods

### Prediction of the siderophore gene clusters

antiSMASH webserver was used to investigate siderophore biosynthetic genes (Version 6.0.1). antiSMASH has been created by Blin et al. (2021) for the discovery of secondary metabolites in complete genome or metabolic gene clusters. The default parameters were used for the antiSMASH analysis with relaxed detection strictness. AntiSMASH known clusters and sub clusters were also investigated in the present study.

### BLASTp analysis

Also, BLASTp webserver was used to find sequences with similar gene regions. BLASTp analysis compares nucleotide or protein sequences to sequence databases and calculates the statistical significance of matches. BLAST can be used to infer functional and evolutionary relationships between sequences and help identify members of gene families (Johnson et al. 2008). BLASTp analysis was performed to search in the non-redundant protein sequences from the NCBI database. The criteria used to determine the sequences according to the BLASTp results were the e-value is ≤ 0.01 and percent identity is ≥ 98%.

### Nucleotide sequence accession numbers

The complete genome sequence of *E. coli* Nissle 1917, *E. coli* K-12 strain MG1655 and *E. coli* VR50 were retrieved from the GenBank (www.ncbi.nlm.nih.gov) database under accession numbers CP007799.1, U00096.3 and CP011134.1, respectively.

## Results

### AntiSMASH analysis of pathogenic *E. coli* VR50 genome

**Fig. 1:**
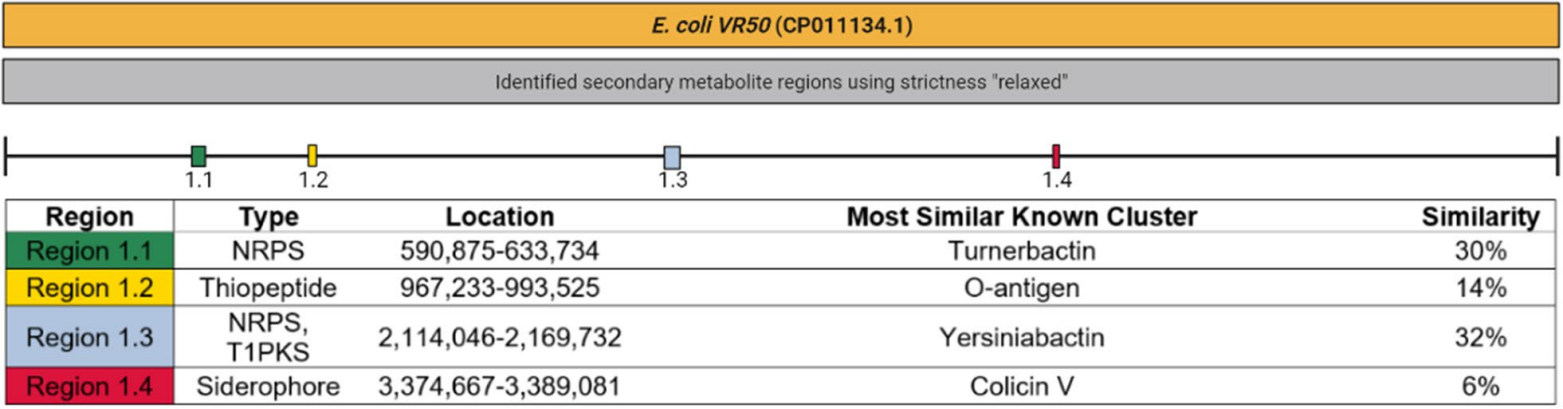
Identified secondary metabolite regions in *E. coli* VR50 (NCBI Accession number: CP011134.1) and similar gene clusters

*E. coli* VR50 has four genomic regions for the biosynthesis of secondary metabolites according to the antiSMASH analysis (Fig. 1). When these regions are studied one-by-one, Region 1.1 was found to be responsible for the biosynthesis of NRPS. NRPS synthesize nonribosomal peptides, which are secondary metabolites produced by bacteria. The NRPS in this region is responsible for enterobactin biosynthesis. Additionally, there are gene clusters related to enterobactin biosynthesis and transport in this region. In Region 1.2, gene clusters responsible for the biosynthesis of thiopeptide were found. Thiopeptides are natural products with antibiotic effects to eliminate competitive microorganisms in the same medium (Chan and Burrows 2021). Region 1.3 contains the NRPS/PKS domain. These domains show NRPS and PKS related functions. Polyketide synthases (PKSs) are proteins or protein complexes that produce a large variety of secondary metabolites found in bacteria, fungi, plants (Weng and Noel 2012). In this region, there are gene clusters responsible for yersiniabactin biosynthesis. Region 1.4 is responsible for the biosynthesis of aerobactin. Additionally, Fig. 1 shows gene clusters containing genes like *E. coli* VR50 genomic regions and their metabolites are shown with similarity rates.

### antiSMASH analysis of nonpathogenic *E. coli* strains

antiSMASH analysis was performed to compare the secondary metabolites of pathogenic *E. coli* VR50 and nonpathogenic *E. coli* Nissle 1917 (O6:K5:H1) and *E. coli* K-12 strain MG1655. Complete genome sequences of *E. coli* Nissle 1917 and *E. coli* K-12 were retrieved from the NCBI database.

**Fig. 2:**
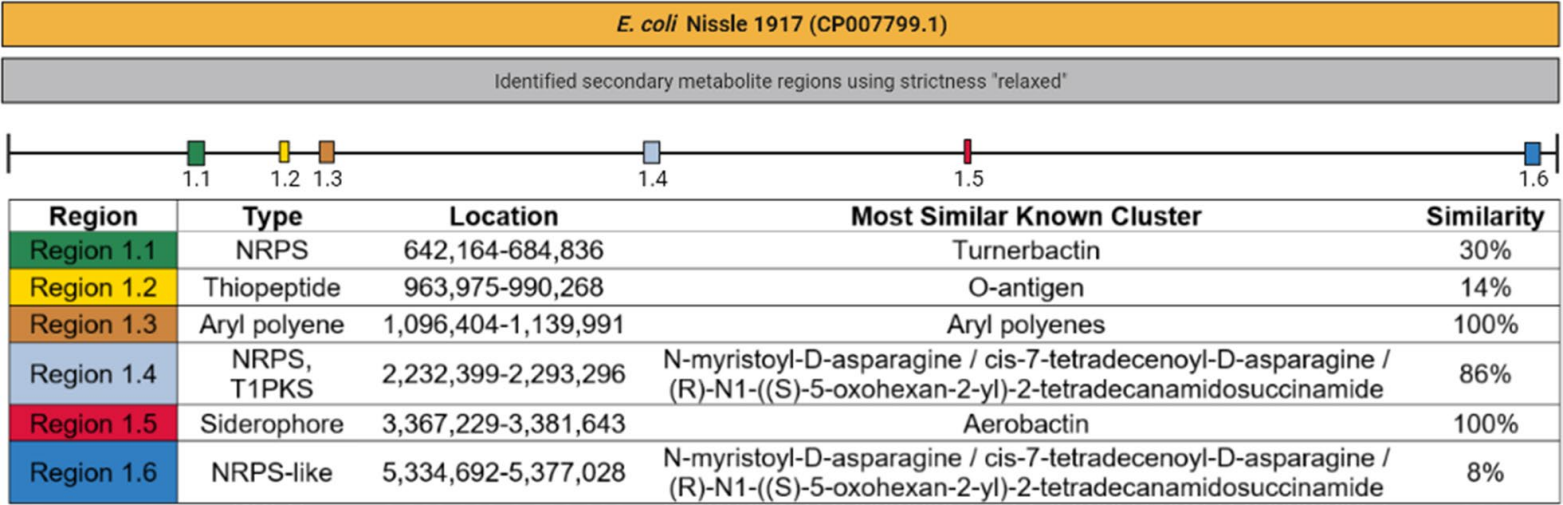
Predicted secondary metabolites in *E. coli* Nissle 1917 (NCBI Accession number: CP007799.1) and similar gen clusters with percentages

As Fig. 2 shows, *E. coli* Nissle 1917 has 6 genomic regions responsible for different secondary metabolites. Region 1.1 synthesizes NRPSs and these NRPSs are responsible for enterobactin biosynthesis and transport. Region 1.2 contains genes for thiopeptide synthesis. Region 1.3 is aryl polyenes biosynthetic gene cluster. Aryl polyenes are polyunsaturated carboxylic acids that can be found in both nonpathogenic and pathogenic strains (Johnston et al. 2021). Region 1.4 is responsible for NRPS and PKS synthesis. There are genes related to aerobactin biosynthesis in Region 1.5. The latter contains the same lysine 6-monooxygenase and siderophore gene clusters (*IucA/IucC).* These gene clusters were also observed in the *E. coli* VR50 genome. Region 1.6 is involved in the synthesis of NRPS-like molecules.

**Fig. 3:**
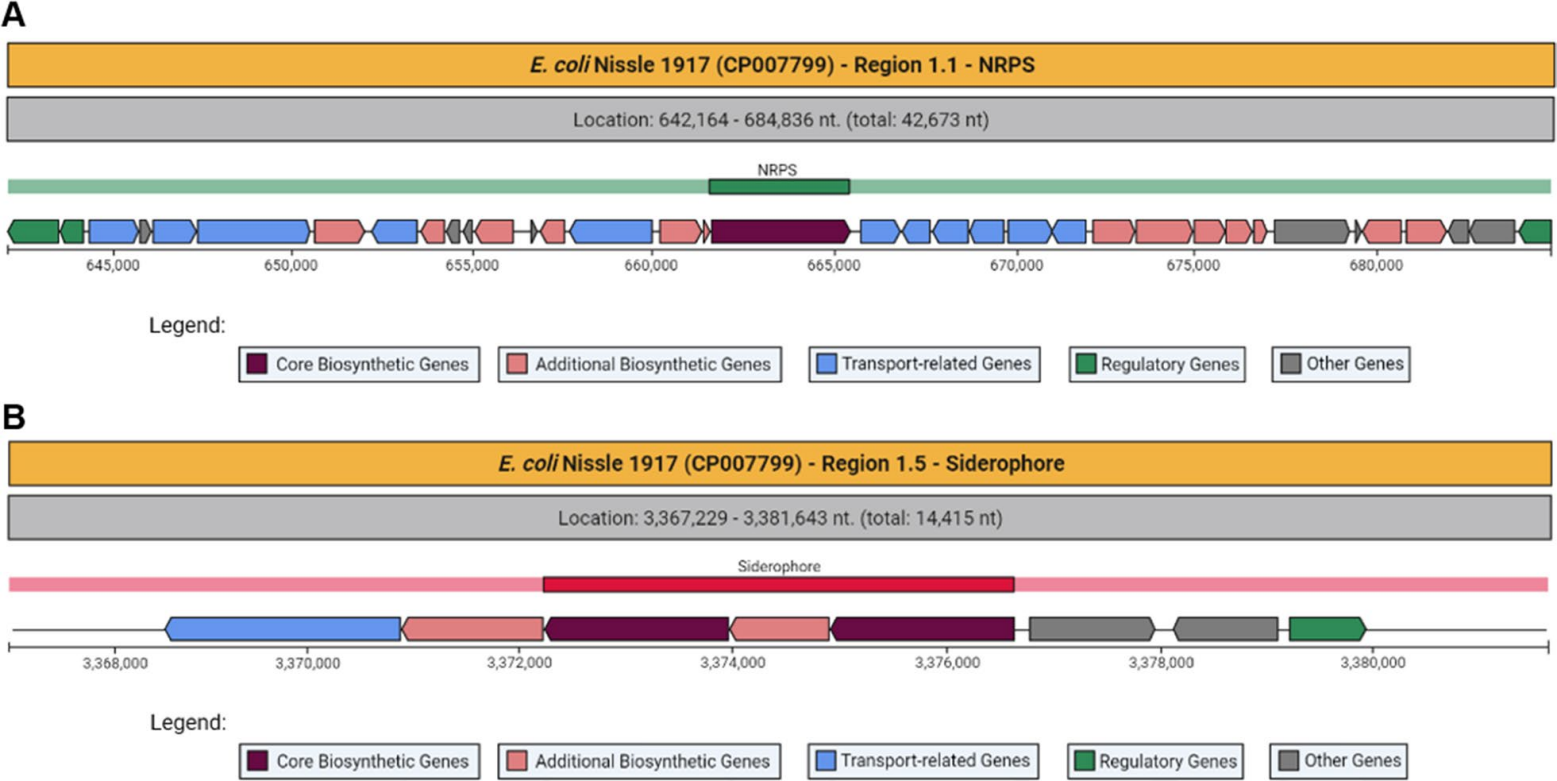
Region 1.1, which is responsible for enterobactin biosynthesis and transport (**A**); Region 1.5 containing aerobactin biosynthetic genes (**B**) in *E. coli* Nissle 1917

In Fig. 3A, enterobactin-related genes are located at 660,180–671,984 nt. Region 1.1 contains TonB-dependent receptor family, enterobactin esterase, enterobactin synthase and six enterobactin transport genes.

As for the Region 1.5 aerobactin biosynthetic region, there are three aerobactin biosynthetic genes, TonB-dependent receptor, and lysine/ornithine N-monooxygenase gene (Fig. 3B).

**Fig. 4:**
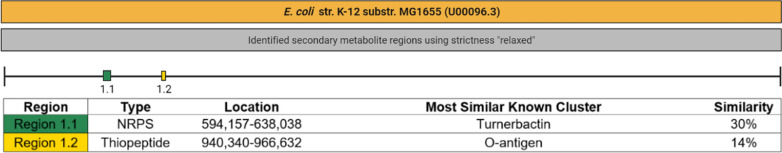
Predicted secondary metabolites in *E. coli* strain K-12 sub strain MG1655 (NCBI Accession number: U00096.3)

**Fig. 5:**
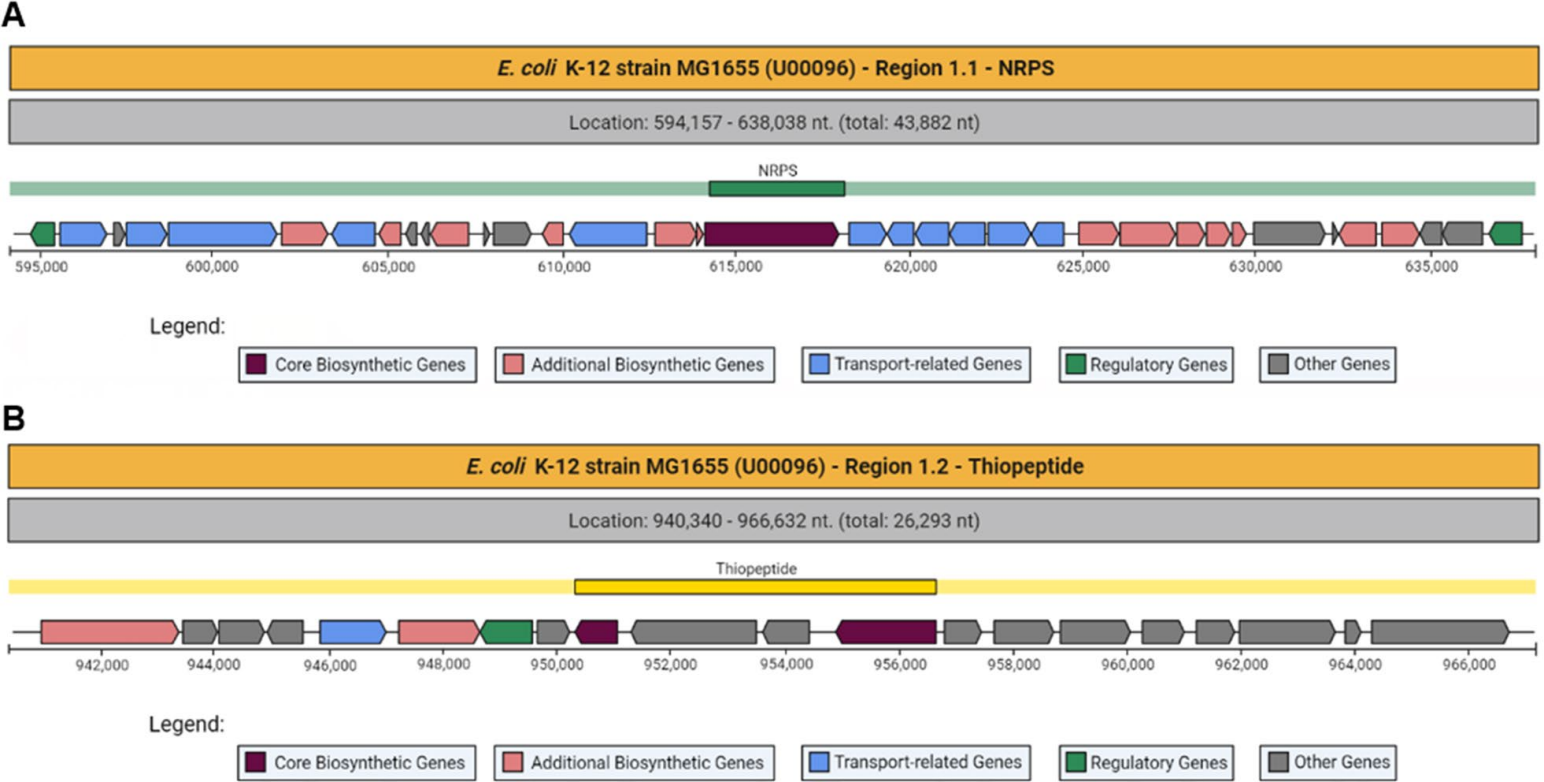
NRPS gene region in the *E. coli* K-12 strain MG1655 (**A**); Thiopeptide gene region (**B**) in the *E. coli* K-12 strain MG1655

There are 2 genomic regions in *E. coli* K-12 strain MG1655. *E. coli* K-12 strain MG1655 does not have a genomic region in its genome that is responsible for NRPS-independent siderophores (Fig. 4). Although when the two genomic regions were examined, genes responsible for enterobactin transport were found in Region 1.1 (Fig. 5A, These regions are blue colored genes between 610,254 and 624,510 nt.). TonB-dependent siderophore receptor family, enterobactin/ferric enterobactin esterase, ferric enterobactin transport proteins (FepE) and ATP-binding protein are the genes responsible for enterobactin transport. TonB-dependent siderophore receptor family and ATP-binding protein are also found in pathogenic *E. coli* VR50 genome.

As can be seen from Fig. 5B, this region has two thiopeptide biosynthetic genes and many putative genes.

### Comprehensive antiSMASH analysis of *E. coli* strains

To see the comparability of the study, 7 pathogenic and 4 nonpathogenic *E. coli* strains were analyzed with the antiSMASH (Table 2). According to the results, it was observed that similar siderophores were produced in different strains. As can be seen in Table 2, nonpathogenic *E. coli* strains synthesize fewer types of siderophores, and among these siderophores, yersiniabactin is less common. On the other hand, all examined UPEC strains contain yersiniabactin. Enterobactin siderophore was found in all pathogenic and nonpathogenic strains.

**Table 2:** Siderophores found in both pathogenic and nonpathogenic *E. coli* strains by antiSMASH analysis

Strain	Accession number	Pathogenicity	Siderophores predicted by antiSMASH	References
*Escherichia coli* UTI89	NC_007946	Pathogenic	Enterobactin, yersiniabactin	Mortensen et al. (2019)
*Escherichia coli* strain CFT073	NZ_CP058222	Pathogenic	Aerobactin, yersiniabactin, enterobactin	Luo et al. (2009)
*Escherichia coli* strain F11	NZ_CP076123	Pathogenic	Yersiniabactin, enterobactin	Koch et al. (2011)
*Escherichia coli* strain 536	NC_008253	Pathogenic	Enterobactin, yersiniabactin	Dobrindt et al. (2010)
*Escherichia coli* strain NA114	NZ_MIPU00000000	Pathogenic	Enterobactin, yersiniabactin, aerobactin	Avasthi et al. (2011)
*Escherichia coli* strain 83,972	NZ_CP058220	Pathogenic	Aerobactin, yersiniabactin, enterobactin	Roos et al. (2006)
*Escherichia coli* O15:K52:H1	NZ_NKDL00000000	Pathogenic	Yersiniabactin, enterobactin, aerobactin	Olesen et al. (2009)
*Escherichia coli* strain C	CP029371	Nonpathogenic	Enterobactin	Hamasha et al. (2013)
*Escherichia coli* strain 1307	NZ_JVUM00000000	Nonpathogenic	Aerobactin, yersiniabactin, enterobactin	Reissbrodt et al. (2009)
*Escherichia coli* strain C600	NZ_CP031214	Nonpathogenic	Enterobactin	Goswami et al. (2015)
*Escherichia coli* strain ATCC 25922	NZ_CP037449	Nonpathogenic	Aerobactin, enterobactin, yersiniabactin	Leenanon and Drake (2001)

### Siderophore genomic region in *E. coli* VR50

**Fig. 6:**
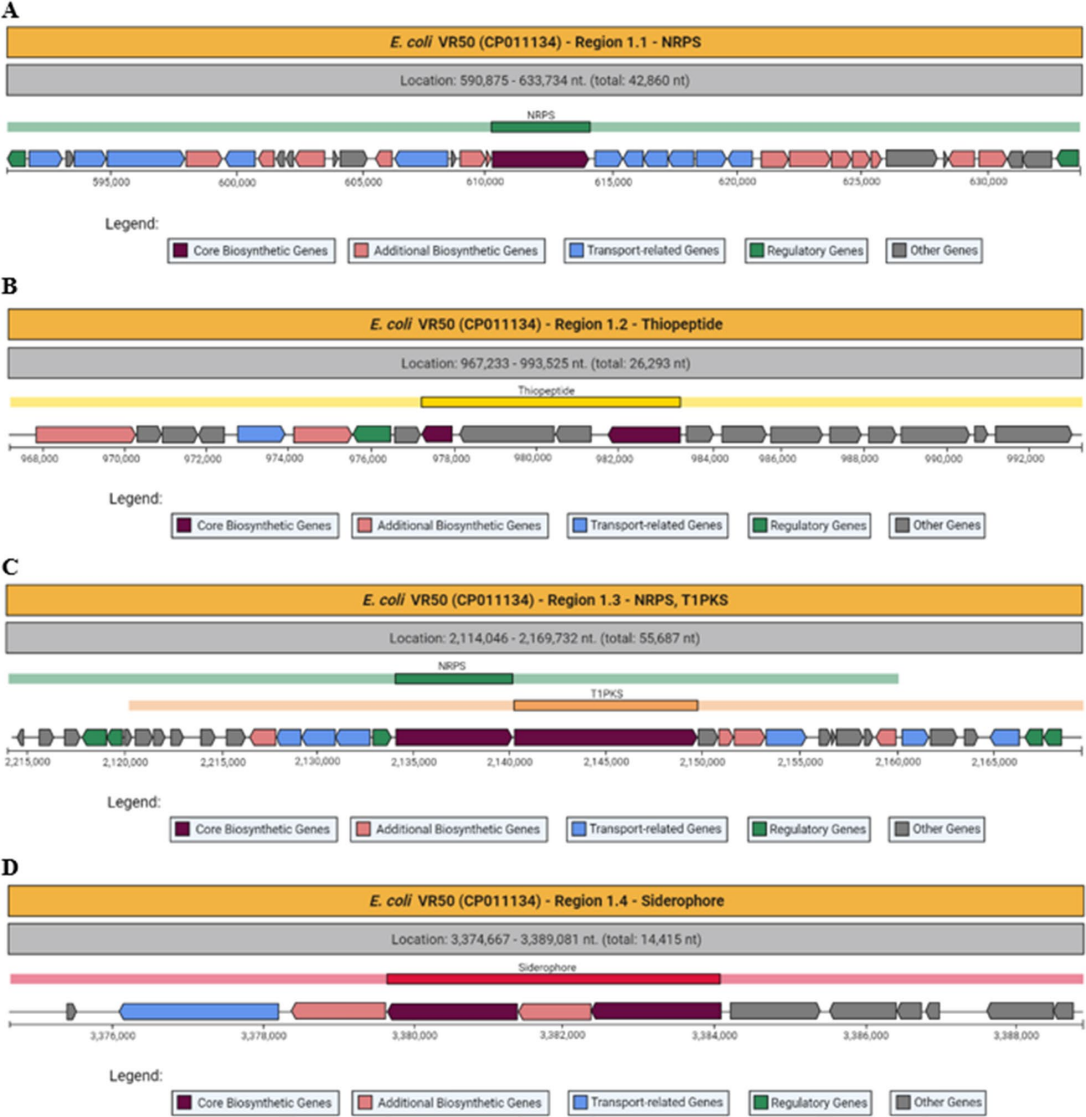
Region 1.1 responsible for the biosynthesis of enterobactin and transport (**A**); Region 1.2 thiopeptide biosynthetic gene cluster (**B**); Region 1.3 responsible for NRPS and PKS genes related to yersiniabactin biosynthesis (**C**); Region 1.4 siderophore biosynthetic gene cluster (**D**) in *E. coli* VR50

Region 1.1 of the *E. coli* VR50 genome contains a core NRPS biosynthetic gene and multiple other genes. There is an enterobactin synthase gene and eight enterobactin transport-related genes (Fig. 6A, These are blue colored genes between 608,930 and 623,796 nt.).

As can be seen from Fig. 6B, Region 1.2 has two thiopeptide biosynthetic genes and many enzymes related genes like dimethyl sulfoxide reductase and transferases.

NRPS and PKS in Region 1.3 are yersiniabactin biosynthetic genes. As we mentioned before, yersiniabactin is the most synthesized siderophore by pathogenic strains. Other than NRPS and PKS regions, there are three yersiniabactin biosynthetic protein genes between 2,149,729 and 2,153,210 nt. and one TonB-dependent receptor gene at 2,153,341–2,155,362 nt (Fig. 6C).

As can be seen from Fig. 6D, there are 12 genes in the Region 1.4 siderophore biosynthetic gene cluster in *E. coli* VR50 due to antiSMASH analysis. This siderophore biosynthetic gene cluster is located at 3,374,667–3,389,081 nt. In this region there are three siderophore biosynthesis protein genes, one *IutA* and one *IucD* gene. These 12 genes contain hypothetical proteins, TonB-dependent siderophore receptor family, lysine/ornithine N-monooxygenase, *IucA/IucC*, putative siderophore biosynthesis protein and transposases (Table 3).

**Table 3:** The genes in the Region 1.4 of *E. coli* VR50 genome

Gene	Domain annotations	Location
Hypothetical protein	Unknown	3,375,431–3,375,559 (Total: 129 nt)
*IutA*	TonB-dependent siderophore receptor family	3,376,110–3,378,254 (Total: 2,145 nt)
L-lysine 6-monooxygenase *IucD*	Lysine/ornithine N-monooxygenase	3,378,393–3,379,670 (Total: 1,278 nt)
Siderophore biosynthesis protein	Siderophore: *IucA/IucC*	3,379,667–3,381,409 (Total: 1,743 nt)
Siderophore biosynthesis protein	Putative siderophore biosynthesis protein	3,381,409–3,382,356 (Total: 948 nt)
Siderophore biosynthesis protein	Siderophore: *IucA/IucC*	3,382,357–3,384,081 (Total: 1,725 nt)
Putative membrane transport protein	Unknown	3,384,217–3,385,410 (Total: 1,194 nt)
IS2 transposase B	Transposase	3,385,523–3,386,428 (Total: 906 nt)
IS2 transposase A	Unknown	3,386,421–3,386,750 (Total: 330 nt)
Hypothetical protein	Unknown	3,386,809–3,386,994 (Total: 186 nt)
IS629 transposase B	Transposase	3,387,603–3,388,493 (Total: 891 nt)
IS629 transposase A	Transposase IS3/IS911 family protein	3,388,490–3,388,765 (Total: 276 nt)

Hypothetical proteins are predicted to be expressed in an organism, but the corresponding translation product has not been characterized yet (Ijaq et al. 2019). IutA is defined as the ferric aerobactin receptor. IutA has been associated with the pathogenicity of UPEC strains (Landgraf et al. 2012). IucD catalysis L-lysine 6-monooxygenase reaction. This enzyme is the initial step of the aerobactin biosynthesis pathway. Aerobactin is a bacterial iron chelating agent found in *E. coli* (Thariath et al. 1993). IucA/IucC is an iron uptake chelate domain that is involved in the biosynthesis of the siderophores (Oves-Costales et al. 2009). Transposases are enzymes that move non-expressed transposon regions to different parts of the genome by cut-and-paste mechanism (Lewis et al. 2004).

### KnownClusterBlast and SubClusterBlast analysis

**Fig. 7:**
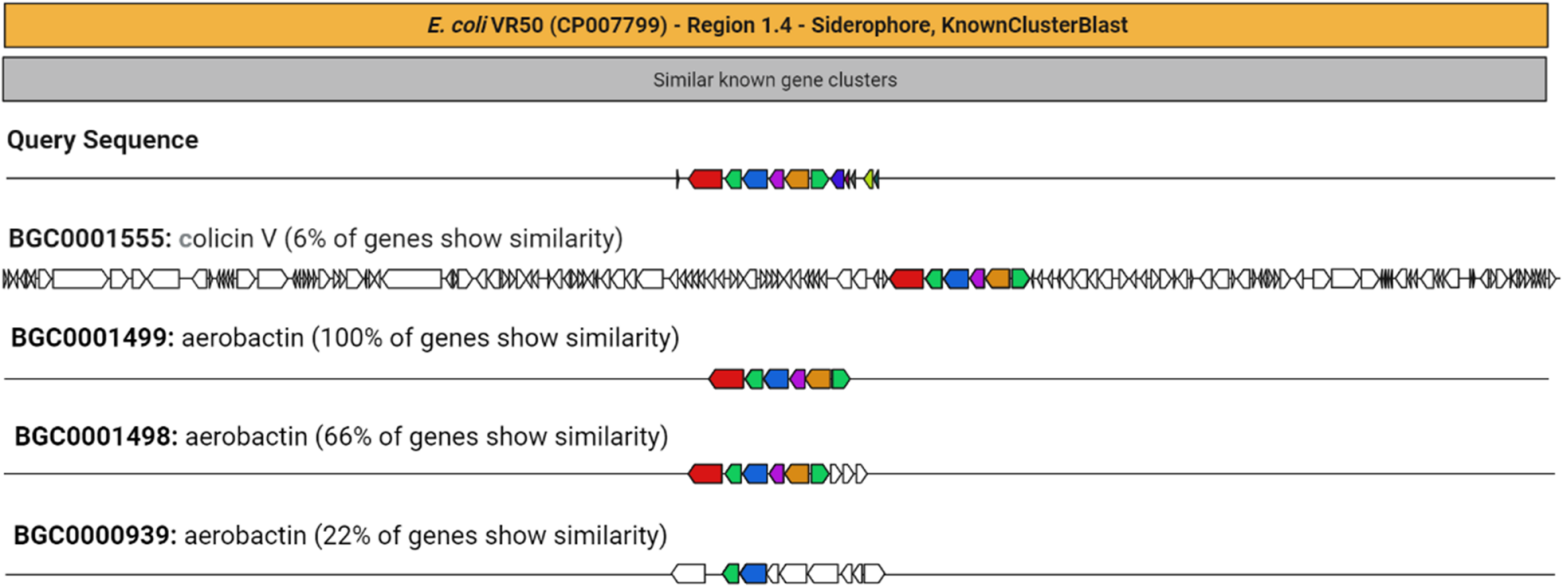
Similar gene clusters compared to *E. coli* VR50 siderophore biosynthesis gene cluster

Figure 7 shows similarity percentages of biosynthetic gene clusters with respect to *E. coli* VR50 siderophore region. These similarities as percentages in colicin V (*E. coli* chi7122, BGC0001555), aerobactin (*Pantoea ananatis,* BGC0001499), aerobactin (*Xenorhabdus szentirmaii* DSM 16,338, BGC0001498) and aerobactin (*Grimontia hollisae,* BGC0000939) were 6%, 100%, 66% and 22%, respectively. No match was found in the SubClusterBlast analysis.

### BLASTp analysis and results

In addition to antiSMASH, BLASTp analysis was performed to identify organisms with similar sequences. Two of the core siderophore biosynthetic genes in Region 1.4 of the *E. coli* VR50 were analyzed with the BLAST webserver. The first gene is between 3,379,667 and 3,381,409 nt. (Total 1,743 nt.) and the second gene is between 3,382,357–3,384,081 nt. (Total 1725 nt.). To decide the DNA sequences after the BLASTp analysis, e-value and percent identities were selected as ≤ 0.01 and ≥ 98%, respectively (Tables 4, 5).

**Table 4:** The microorganisms showing similar sequences to the first siderophore biosynthetic gene in *E. coli* VR50

Sequence	Organism	Accession number	E-value	Percent identity
NIS family aerobactin synthetase *IucC*	*Enterobacteriaceae*	WP_001015715.1	0.0	100.00%
NIS family aerobactin synthetase *IucC*	*Enterobacteriaceae*	WP_001015713.1	0.0	99.31%
NIS family aerobactin synthetase *IucC*	*Escherichia coli*	WP_063268075.1	0.0	99.83%
Aerobactin synthase *IucC*	*Escherichia coli*	NJY60833.1	0.0	99.83%
TPA: aerobactin synthase *IucC*	*Escherichia coli*	HAG9679402.1	0.0	99.83%
*IucA/IucC* family siderophore biosynthesis protein	*Shigella flexneri*	EFX8038752.1	0.0	99.14%
Aerobactin synthase *IucC*	*Shigella flexneri*	EFZ8886218.1	0.0	99.14%

**Table 5:** The microorganisms showing similar sequences to the second siderophore biosynthetic gene in *E. coli* VR50

Sequence	Organism	Accession number	E-value	Percent identity
NIS family aerobactin synthetase *IucA*	*Enterobacteriaceae*	WP_001296374.1	0.0	100.00%
NIS family aerobactin synthetase *IucA*	*Enterobacteriaceae*	WP_002431271.1	0.0	99.83%
*IucA/IucC* family siderophore biosynthesis protein	*Escherichia coli*	EAB6864461.1	0.0	100.00%
TPA: aerobactin synthase *IucA*	*Escherichia coli*	HAG8887898.1	0.0	99.83%
Aerobactin synthase *IucA*	*Shigella flexneri*	EGE4460618.1	0.0	99.83%
Putative siderophore synthetase component	*Shigella flexneri* 2,002,017	ADA76020.1	0.0	99.83%
aerobactin synthase *IucA*	*Shigella sonnei*	AMG17639.2	0.0	99.83%
*IucA/IucC* family siderophore biosynthesis protein	*Shigella sonnei*	EGD4870342.1	0.0	99.65%

To compare with the data in Tables 4, 5 the effect of microorganisms on UTIs was investigated. *Enterobacteriaceae* are pathogens responsible for pneumonia, UTIs and sepsis (Zilberberg et al. 2017). Carbapenem-resistant *Enterobacteriaceae* (CRE) especially cause major health problems. It is very difficult to treat, as CREs are not affected by the carbapenem antibiotic thanks to the carbapenemase enzyme they produce (Eshetie et al. 2015). There are few reports of *Shigella flexneri* causing UTIs and UTIs caused by *Shigella sonnei* are very unusual. It is not known exactly how *Shigella* species infect the urinary tract (Papasian et al. 1995). However, *Shigella spp.* and *E. coli* are similar in terms of phenotype and genotype. Therefore, it is thought that the virulence factors of *E. coli* may also be present in *Shigella* species (Tufon et al. 2020).

## Discussion

The relation between the siderophore synthesized by the bacteria and its pathogenicity has been shown in previous studies (Feldmann et al. 2007; Henderson et al. 2009; Holden and Bachman 2015). It was found that *E. coli* VR50, whose genome was analyzed, contains gene regions responsible for enterobactin and yersiniabactin biosynthesis. Based on this, it can be concluded that this strain causes UTIs by the effect of these siderophores. Also, nonpathogenic *E. coli* Nissle 1917 contains enterobactin and aerobactin biosynthetic gene regions. It is very interesting that yersiniabactin was existed in pathogenic *E. coli* VR50 but was not found in nonpathogenic *E. coli* Nissle 1917. Moreover, nonpathogenic *E. coli* K-12 strain MG1655 does not contain any gene cluster related to yersiniabactin and aerobactin.

Some *E. coli* strains infect the urinary tract and colonize there, causing UTIs (Kot 2019). One of these strains, *E. coli* VR50’s genome was analyzed to understand how it causes infection. When the genome of *E. coli* VR50 was examined, it was observed that it synthesizes some secondary metabolites that could ensure its survival in the urinary tract. The presence of siderophores among these metabolites attracted our attention. Although siderophore biosynthetic and siderophore-related genes are found in both pathogenic and nonpathogenic strains, they affect the pathogenicity of a strain. In the absence of siderophores, UPEC strains cause low-level local symptoms (Holden and Bachman 2015).

By inhibiting the iron uptake pathways, the pathogenicity of the microorganism can be reduced. Some of the studies focused on the reduction or inhibiting these metabolic pathways are shortly reviewed here. Chelating agents with higher iron affinity than siderophores was targeted to reduce the iron uptake of the microorganism by Qiu et al. (2011). The authors found that iron (III)-selective 3-hydroxypyridin-4-one chelator with very high affinity for iron shows antimicrobial effect and this compound is proposed to treat open wounds. This indicates that the bacterial cell wall structure and physical properties of the chelating agent are also important in inhibiting bacterial growth. Coulanges et al. (1997) conducted some studies on the strain *Listeria monocytogenes* which is known not to produce siderophores but uses external siderophores for iron uptake. In the study, it was found that Pt (II) was an inhibitor for the ferric reductase enzyme found in *L. monocytogenes*. Iron uptake was completely inhibited after treating the microorganism with Pt (II). Thus, ferric reductase activity was found to be fundamental for bacterial iron uptake. Schalk (2018) reported that antibiotics can be attached to siderophores and transport new antibiotics into bacteria using the iron intake mechanism. In the research of Schalk (2018), siderophores are used as a “*Trojan Horse*” transporting antibiotics into bacteria without being detected. Since iron is found in hemoglobin, it is responsible for oxygen transport in humans. However, excess iron increases the risk of cancer. Cancer cells need more nutrients than normal cells because they multiply continuously and rapidly. Since iron is necessary for growth, tumor cells require more iron. Considering this situation, siderophores, which are small iron chelators, are used for treating cancer (Saha et al. 2016). It is very interesting to note that siderophore production in *E. coli* strains isolated from wild- and factory-raised turkeys are different, reported by Craft et al. (2022). Also, it was stated in the study that *E. coli* strains isolated from factory-raised turkeys produce more siderophores and other virulence factors compared to wild types. Siderophores are not specific for *E. coli* strains, these molecules are also reported from various species for many different actions. Some of the recent papers are reviewed here. In an interesting study carried out by Shah et al. (2022), the production of siderophores was investigated from five endophytic bacterial strains of the Pakistani wheat varieties. Endophytic bacteria are recently considered as a support for the plant productivity and defense system. Shah et al. (2022) found out that some of the strains have an ability to synthesize siderophores which could be associated with growth-promoting effects for the commercial plants. Podgórska-Kryszczuk et al. (2022) also studied biological control of pathogenic *Fusarium* spp. by using antagonistic yeasts. The production of siderophores is also underlined by the authors as one of the action mechanisms against these species. Roskova et al. (2022) studied the use of siderophores as a bioremediation tool. Although the main role of siderophores is to bind iron, they can also chelate other heavy metals. This feature is of great importance in the solubilization and transport of heavy metals in the soil. Bioremediation methods are being developed by using microorganisms and plants that synthesize siderophores. Although the pathogenicity of some microorganisms has been known for a long time, it has been found later that they synthesize siderophores (Courcol et al. 1997). Comprehensive studies on *E. coli* VR50 should be conducted to give a more precise information about the pathogenicity. Therefore, new studies should be carried out on siderophores and their pathogenic properties.

The studies were found to show that the results obtained in antiSMASH analyzes were compatible with laboratory results. Lv et al. (2014) studied with non-pathogenic K12 strain MG1655 and uropathogenic UTI89 to reveal which kinds of siderophores they synthesize. Stable isotope dilution LC–MS/MS method is used to quantify the siderophores. According to their result, both strains produce enterobactin and uropathogenic UTI89 also produces yersiniabactin and salmochelin. Porcheron et al. (2014) studied the roles of iron regulators RhyB and Fur in the UPEC strain CFT073. Different amounts of siderophores were synthesized with deletion of RhyB and Fur genes. As a result of the study, it was found that aerobactin, enterobactin, and salmochelin siderophores were synthesized in a decreasing manner in the unmutated CFT073 strain. Garcia et al. (2011) conducted a study about the effect of outer membrane iron receptors in urinary tract infections. When the mutated strains were examined, they found that some siderophores were more effective in urinary tract colonization. When wild-type strains were examined enterobactin, salmochelin, aerobactin were found in *E. coli* CFT053 and enterobactin, salmochelin, yersiniabactin were found in *E. coli* 536. Cui et al. (2022) developed an indirect competitive ELISA (ic-ELISA) for enterobactin quantification. The ic-ELISA can detect enterobactin in different microorganisms. *E. coli* AN102, *E. coli* ATCC 25922, *Salmonella enteritidis* CVCC 1806, *Campylobacter jejuni* NCTC 11168, *Bacillus cereus* ATCC 14579, and *Staphylococcus aureus* ATCC 29213 strains were grown in iron-restricted medium. Results show that, *E. coli* AN102, *E. coli* ATCC 25922, and *S. enteritidis* CVCC 1806 can produce enterobactin, while other strains cannot. The effect of siderophores produced by *E. coli* strain 83972 on urinary tract infections was studied by Watts et al. (2012). Siderophores synthesized by *E. coli* strain 83972 were determined by HPLC/LC–MS analysis. As a result of the analysis, it was found that *E. coli* 83972 produces the enterobactin, salmochelin, aerobactin, and yersiniabactin siderophores.

A literature search reveal that some of the strains that were analyzed in Table 2 also contains salmochelin siderophore, but in antiSMASH analysis salmochelin gene clusters were absent. Salmochelin is an enterobactin that is C-glycosylated. Salmochelin is produced by *Salmonella* species and some UPEC strains (Hantke et al. 2003). IroN, IroD and IroB genes in *Salmonella* species are similar to the enterobactin receptor FepA, enterobactin esterase Fes, and glycosyltransferases, respectively (Müller et al. 2009). Bister et al. (2004) found that IroB gene is responsible for the glycosylation of enterobactin.

In summary, secondary metabolites produced by *E. coli* VR50, which causes UTIs, were examined. Siderophores have been found to be effective in the pathogenicity of the bacteria. However, its main role in infection varies according to the type and amount of siderophore.

In conclusion, genome mining tools which have been developed under in silico methodologies provide big contributions to understanding the etiology of the diseases. Moreover, identification and suppression of specific genes in pathogenic microorganisms that are responsible for secondary metabolites could be a new gate in the therapy of diseases that are based on pathogenic microorganisms. By using recombinant DNA technology, siderophore-based molecules can be expressed in recombinant bacteria for agriculture and other industrial processes.

## Data Availability

The data presented in this study are available on request from the corresponding author.
